# Association Use of Bisphosphonates with Risk of Breast Cancer: A Meta-Analysis

**DOI:** 10.1155/2020/5606573

**Published:** 2020-10-06

**Authors:** Rui Peng, Xingzhong Liang, Gang Zhang, Yuan Yao, Zhen Chen, Xiaojuan Pan, Jinshan Wang, Genglong Liu

**Affiliations:** ^1^Breast Surgery, Xuzhou Maternity and Child Health Care Hospital, Xuzhou, 221000 Jiangsu Province, China; ^2^Department of Intensive Care Unit, Shunde Hospital, Southern Medical University (The First People's Hospital of Shunde), Foshan, 528308 Guangdong Province, China; ^3^Department of General Medicine, The First Affiliated Hospital, Sun Yat-sen University, Guangzhou, 510080 Guangdong Province, China; ^4^Department of Neurosurgery, The First Affiliated Hospital, Sun Yat-sen University, Guangzhou, 510080 Guangdong Province, China; ^5^Department of Pathology, Affiliated Cancer Hospital & Institute of Guangzhou Medical University, Guangzhou, 510095 Guangdong Province, China

## Abstract

**Background:**

Previous studies have investigated the association between the use of bisphosphonates and the development of breast cancer, which presented controversial results. Thus, this meta-analysis was conducted to summarize the current evidence of the association of bisphosphonate use with breast cancer risk.

**Methods:**

A comprehensive search was conducted in PubMed, ISI Web of Knowledge, the Cochrane Library, and Embase from inception to March 2019 by two researches, who independently selected trials, retrieved relevant data, and assessed study quality. The summary relative risk (RR) for the use of bisphosphonates on the risks of developing breast cancer was calculated using a random-effect model.

**Results:**

The present meta-analysis, which included four case-control studies, involving 55052 breast cancer cases, and seven retrospective cohort studies, involving 14641 breast cancer cases, assessed the effect of bisphosphonates on breast cancer risk. The random-effect model meta-analysis found a reduced risk of breast cancer with exposure to bisphosphonates with pooled RR of 0.87 (95% confidence interval [CI]: 0.80 to 0.94). The short-term use of bisphosphonates (<1 year) did not render significant alteration (RR = 0.92, 95% CI: 0.82 to 1.03), while a significant 26% risk reduction of breast cancer was noted with long-term use (>1 year) (RR = 0.74, 95% CI: 0.62 to 0.90). A protective effect of bisphosphonates was shown in contralateral breast cancer (RR = 0.41, 95% CI: 0.20 to 0.84). In terms of the type of bisphosphonates, a significant inverse relationship was noted for etidronate, with pooled RR of 0.87 (95% CI: 0.80 to 0.96).

**Conclusion:**

This meta-analysis suggested that the use of bisphosphonates was associated with reduced risk of breast cancer, including contralateral breast cancer. Compared to other types of bisphosphonates, only etidronate showed a significant inverse relationship. Additionally, the long-term use (>1 year) of bisphosphonates was more significant in lowering breast cancer risk. Further randomized controlled trials are needed to verify this association. This trial is registered with PROSPERO (registration number: CRD42018105024) (registered on 29 August 2018).

## 1. Introduction

Bisphosphonates are extensively used to treat osteoporosis, as well as prevent and treat skeletal destructive lesions caused by malignancy, particularly among postmenopausal women [1, 2]. Several studies in vitro have shown that bisphosphonates have a series of direct and indirect antitumor effects, such as induction of tumor cell apoptosis, prevention of tumor adhesion and invasion, as well as inhibition of tumor angiogenesis and cell proliferation [3]. In addition, preclinical studies have found that bisphosphonates can induce breast carcinoma cell death and inhibit estrogen-sensitive MCF-7 cell proliferation [4, 5], which make bisphosphonates an attractive class of drugs to be studied further for breast cancer prevention.

Numerous epidemiologic studies have investigated a relationship between bisphosphonate use and breast cancer risk [6–16]. For instance, the risk of invasive breast cancer in women prescribed as bisphosphonates for treatment of osteoporosis was reduced by 32% after adjustment for potential confounders in the Women's Health Initiative (WHI) observation study [8]. On the contrary, the Chiang et al. study [7], using the National Health Insurance Research Database (NHIRD), found that bisphosphonates did not lower the risk of breast cancer, with the limited study size and underpowered results. Additionally, two peer-reviewed meta-analyses published consistently reported that use of bisphosphonates was associated with a reduced risk of breast cancer [17, 18].

However, subsequent publications of two large studies have showed opposite results [9, 10], and so far, researches as well as systematic reviews have not addressed whether bisphosphonate use can decrease the risk of breast cancer. The ongoing debate has given renewed impetus by recent studies with no clear conclusions. Hence, to provide an up-to-date evidence summary, we performed a comprehensive meta-analysis to summarize all current studies to precisely quantify whether use of bisphosphonates is associated with the risk reduction of breast cancer and to explore the different types of bisphosphonate effects on breast cancer, duration of exposure to bisphosphonates on breast cancer risk, and effects of bisphosphonates on different types of breast cancer.

## 2. Methods

This meta-analysis followed the Meta-Analysis of Observational Studies in Epidemiology (MOOSE) [19]. The review protocol was registered in the international prospective register of systematic reviews in August 2018 (PROSPERO registration number: CRD42018105024).

### 2.1. Literature Retrieval

We systematically searched from inception to March 2019, using the PubMed, ISI Web of Knowledge, Cochrane Library, and Embase databases. All relevant published articles about human subjects were identified using the following terms: (breast or mammary gland), (neoplasm∗or tumor∗or cancer∗or carcinoma∗), and (diphosphonate∗or bisphosphonate∗or alendronate∗or etidronate∗or clodronateor∗or zoledronate∗or risedronate∗or ibandronate∗or pamidronate∗or tiludronate∗). No publication date or language restrictions were adopted. In addition, a manual search of references cited in the selected articles and published reviews on the same topic was conducted to obtain any additional relevant trials.

### 2.2. Eligibility Criteria

Using standard forms, two authors independently assessed the trials for candidates based on the inclusion and exclusion criteria in the meta-analysis. In cases of divergent views, a consensus was reached through discussion or in consultation with the third author. Studies were considered eligible if they (1) focused on the association between bisphosphonate use and breast cancer risk; (2) reported relative risk (RR), odds ratio (OR), or hazard ratio (HR) with 95% CI (or sufficient data to compute these); and (3) included a trial with the most relevant information with respect to repetitive trials. The exclusion criteria were review articles, case reports, letters to the editor, editorials, and if the outcome of interest was not reported, even after contacting the corresponding authors.

### 2.3. Data Extraction

Data was abstracted independently by two reviewers, with inconformity addressed by consensus and discussion with a third reviewer. The following information was collected from each included trial, such as the first author, year of publication, nation, study design, period, study database, age of participants, menopausal status, number of breast cancer cases, number of participants, prevalence of breast cancer, type of breast cancer, type of bisphosphonates, definition of exposure, measure of bisphosphonates use, average exposure period, adjustment factors in multivariate analysis, and risk estimates with 95% CI. We collected the OR (or HR) in the main analysis that adjusted the greatest degree of potential covariates. If possible, outcome data were separately divided into each subgroup. According to the number of breast cancer cases and the number of subjects being lower or higher than the median of the entire studies, they were classified as the “low” or “high” subgroup, respectively. The primary outcome was the relative risk for usage of bisphosphonates and the incidence of breast cancer.

### 2.4. Quality Assessment

The Newcastle-Ottawa Scale (NOS) was used to evaluate the quality of the cohort studies [20], in which the quality of a trial was adjudicated from three broad perspectives: selection, comparability, and exposure/outcome, with four items (one star each), one item (up to two stars), and three items(one star each), respectively. In this analysis, studies were judged as high quality if they scored more than 7 stars.

### 2.5. Statistical Analyses

The primary summary estimate of interest was the adjusted RR for the incidence of breast cancer with bisphosphonate use. Based on adjusted RR estimates and 95% CI, we generate overall estimates for the effect of bisphosphonates on breast cancer by using a DerSimonian-Laird random-effect model. Compared to the fixed-effect model, the random-effect model is more robust due to incorporating into the weighing scheme both within-study and between-study variations [21]. Given that the absolute risk of breast cancer was very low, the OR or HR was equivalent to the RR directly [22]. Furthermore, we conducted risk-stratification analysis for the type of bisphosphonates (alendronate, etidronate, risedronate, clodronate, and zoledronic acid), duration of bisphosphonate use (<1 year of use, 1 to 3 years of use, 3 to 5 years of use, and >5 years of use), type of breast cancer (breast cancer, invasive breast cancer, ductal carcinoma in situ (DCIS), and contralateral breast cancer),

We assessed statistical heterogeneity among studies by using the Cochran *Q* statistic and quantified by *I*^2^ statistics (*P* > 0.10 or *I*^2^ > 50% considered significant heterogeneity). Sensitivity analysis was conducted by eliminating each study at a time to estimate its influence on incorporative RR in the meta-analysis. Additionally, sensitivity analysis was also investigated by removing studies with characteristics different from the others.

To assess whether the association between incidence of breast cancer and bisphosphonate use was modified by clinical parameters, we also did prior-specified subgroups based on study location (Western country vs. Eastern country), study design (cohort vs. case-control), menopausal status (premenopausal and postmenopausal vs. postmenopausal), measurement of bisphosphonate use (self-report vs. medical record), study quality (high quality vs. low quality), number of breast cancer cases (>1000 vs. ≤1000), and number of participants (>20000 vs. ≤20000). Analysis was conducted to evaluate whether the difference was statistically significant between the subgroups. Additionally, subgroup differences were tested by chi-square test—that is, whether the observed differences are aligned with chance alone within the subgroups. A low *P* value or a large chi-square statistic relative to its degree of freedom constructs evidence of heterogeneity beyond chance.

Metaregression analysis (MRA) was conducted to investigate the potential impacts of heterogeneity and confounders on outcomes. Publication year, average age, number of breast cancer cases, number of participants, prevalence of breast cancer, and average exposure period were considered as variables. We assessed publication bias for primary outcomes by funnel plot inspection, in which 10 or more studies provided data.

All meta-analyses were conducted with RevMan version 5.3 (The Cochrane Collaboration, Copenhagen, Denmark) and Stata version 14 (StataCorp, College Station, Texas, USA). All *P* values are double-tailed, and the statistical significance was set at 0.05.

## 3. Results

### 3.1. Identification of Studies

**Figure 1:**
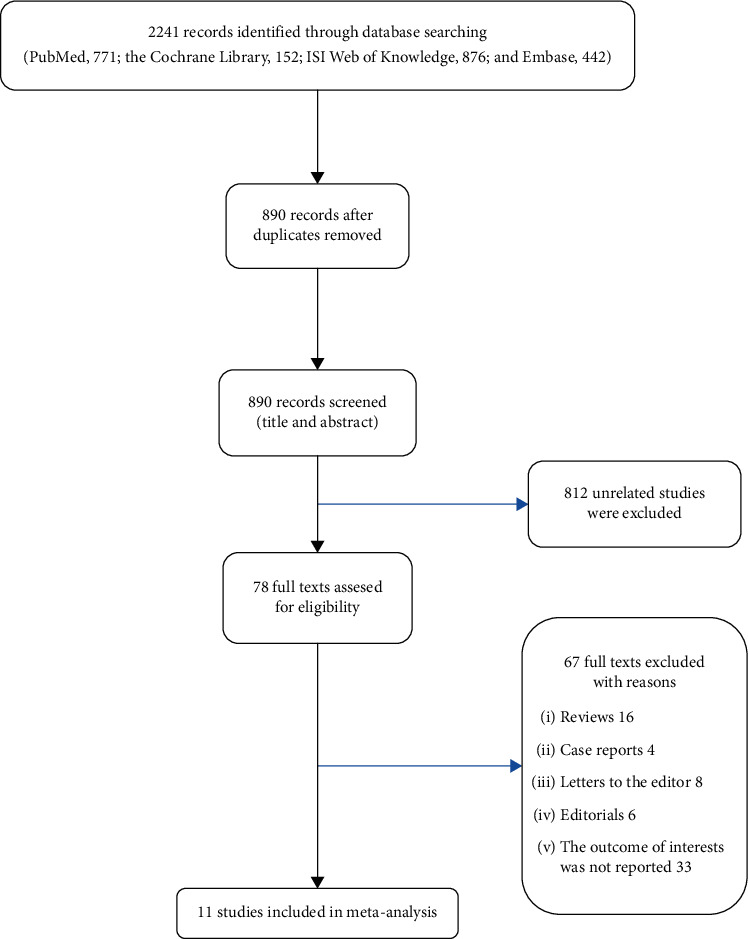
Flow diagram of literature search and selection process of the studies.

The flow chart of the literature selection procedure is shown in Figure 1. The preliminary search identified 771 studies in PubMed, 876 in the ISI Web of Knowledge, 152 in the Cochrane Library, and 442 in Embase. The title and abstract of the remaining 890 articles were screened after omitting 1351 duplicates. After reading their titles and abstracts, 812 studies were eliminated and 78 studies were scrutinized by screening their full texts. Ultimately, 11 studies [6–16] comprising 983807 individuals met the inclusion criteria as well as exclusion criteria which were analyzed.

### 3.2. Study Characteristics and Quality Assessment

A total of eleven studies, including 69693 women with breast cancer, were published between 2010 and 2017, focusing on bisphosphonates and the risk of breast cancer.  Table 1 shows in detail the study characteristics. There were four case-control studies and seven retrospective cohort studies. The studies were performed in Western countries (e.g., UK, USA, France, Israel, and Denmark) or an eastern country (e.g., Taiwan). Six studies involved both premenopausal and postmenopausal women, and five studies recruited only postmenopausal women. The mean age of participants in the eligible studies varied from 54.2 to 73.5 years, while three studies did not report. The number of cases per study varied between 65 and 49933, the number of participants ranged from 1013 to 154768, and prevalence of breast cancer ranged from 0.35 to 3.73%. Additionally, Table 2 details bisphosphonate use of the studies included in the meta-analysis. With respect to types of breast cancer, three studies reported the risk of invasive breast cancer, two studies focused on the risk of DCIS, and one study investigated the risk of contralateral breast cancer. For types of bisphosphonates, alendronate was the most widely used bisphosphonate in all studies. In terms of the measurement of the use of bisphosphonates, three studies and eight studies were based on participants' self-reports and medical records, respectively. Almost all studies used adequate matches or adjustments to control for potential confounders, except for one which only adjusted for age. According to the NOS guideline, the study quality scores of the eleven eligible studies ranged from 6 to 8 (Table S1).

### 3.3. Meta-Analysis

**Figure 2:**
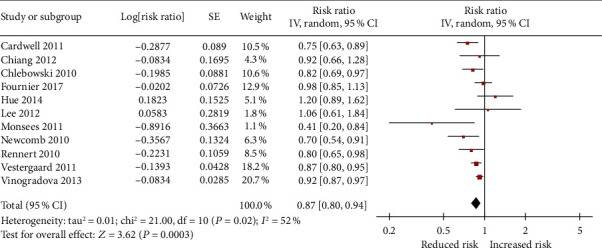
Forrest plot showing the overall effect of bisphosphonates on incidence of breast cancer.

Figure 2 shows the adjusted RR for risk of breast cancer for each trial and all studies pooled comparing any usage of bisphosphonates with no exposure to bisphosphonates. The pooled RR for any usage of bisphosphonates and risk of breast cancer was 0.87 (95% CI: 0.80 to 0.94). Based on *P* = 0.02 and *I*^2^ = 52%, heterogeneity existed between the studies. In a sensitivity analysis, after removing one study by Monsees et al. [12], the heterogeneity of the pooled RR (0.88, 95% CI: 0.82 to 0.94; *P* = 0.06 and *I*^2^ = 45%) showed a relative decrease from high to moderate heterogeneity (Figure S1). Given a study by Lee et al. [11] with inadequately adjusted covariates, a sensitivity analysis in which the studies by Lee et al. were excluded showed a pooled RR of 0.86 (95% CI: 0.80 to 0.94) (Table 3).

**Figure 3:**
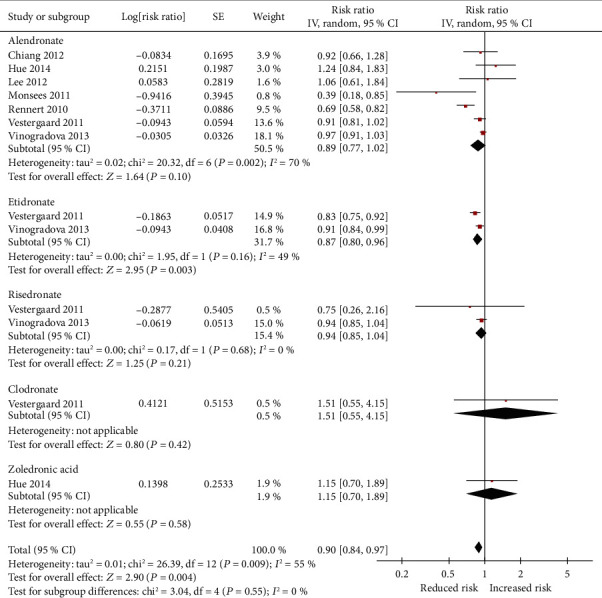
Pooled relative risk (RR) of breast cancer use with type of bisphosphonates.

**Figure 4:**
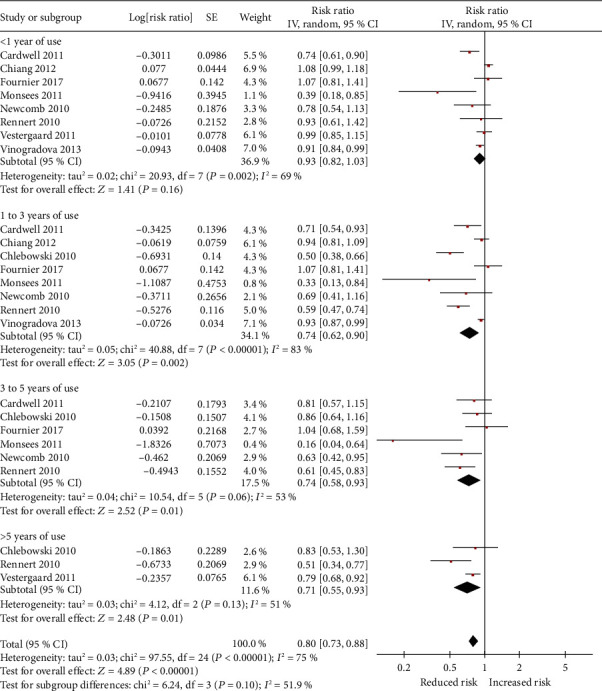
Forest plot for association between duration of the use of bisphosphonates in relation to breast cancer.

**Figure 5:**
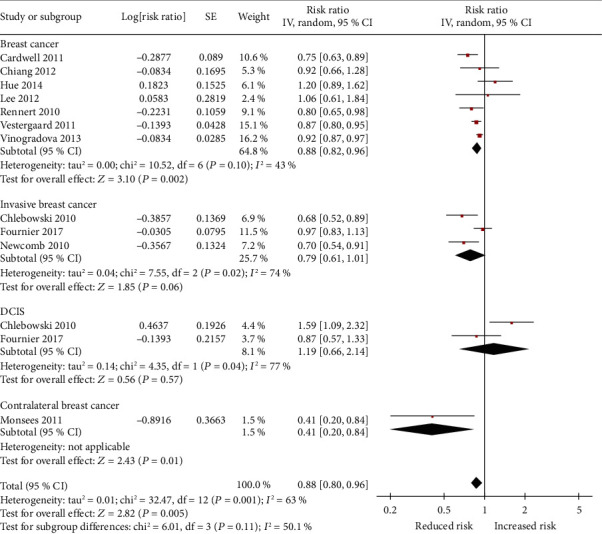
Forest plot showing combined estimates of bisphosphonate use and risk of breast cancer type.

In specific types of bisphosphonate analysis, only etidronate (RR = 0.87, 95% CI: 0.80 to 0.96) could decrease the risk of breast cancer. Nevertheless, alendronate (RR = 0.89, 95% CI: 0.77 to 1.02), risedronate (RR = 0.94; 95% CI: 0.85 to 1.04), clodronate (RR = 1.51, 95% CI: 0.55 to 4.15), and zoledronic acid (RR = 1.15, 95% CI: 0.70 to 1.89) did not render any significant risk reduction in the incidence of breast cancer (Figure 3).  Figure 4 presents the adjusted RR for each trial and combined RR for categories of less than 1 year of use, 1 to 3 years of use, 3 to 5 years of use, and more than 5 years of use. Less than 1 year of use of bisphosphonates was not associated with a reduction in the risk of breast cancer (RR = 0.92, 95% CI: 0.82 to 1.03). A significant inverse association was observed with 1 to 3 years of use (RR = 0.74, 95% CI: 0.62 to 0.90), 3 to 5 years of use (RR = 0.74, 95% CI: 0.58 to 0.93), and more than 5 years of use (RR = 0.71, 95% CI: 0.55 to 0.93). In the risk-stratification analysis within the type of breast cancer, we noted that the usage of bisphosphonates led to a statistically significant reduction risk for any breast cancer (RR = 0.88, 95% CI: 0.82 to 0.96) as well as in contralateral breast cancer (RR = 0.41, 95% CI: 0.20 to 0.84) (Figure 5), whereas the invasive breast cancer subgroup (RR = 0.79, 95% CI: 0.61 to 1.01) and DCIS subgroup (RR = 1.19, 95% CI: 0.66 to 2.14) did not find significant risk reduction.

### 3.4. Subgroup Analyses of Breast Cancer Risk

Table 3 shows the subgroup analyses for the association between any bisphosphonate use and the risk of breast cancer. Subgroup analyses showed that the results were generally consistent, in spite of the study design. However, other subgroup analyses presented that the results were incompatible. In the Western countries, the pooled RR was 0.86 (95% CI: 0.79 to 0.94), but in the Eastern country, there was no significant association (RR = 0.96, 95% CI: 0.71 to 1.27). A significantly protective effect of use of bisphosphonates was presented in premenopausal and postmenopausal women (RR = 0.83, 95% CI: 0.75 to 0.93) yet not in postmenopausal women (RR = 0.92, 95% CI: 0.81 to 1.04). The significant risk reduction for breast cancer after usage of bisphosphonates was also presented in the medical record subgroup (RR = 0.87, 95% CI: 0.80 to 0.96), in the high quality subgroup (RR = 0.88, 95% CI: 0.82 to 0.93), in more than 1000 cases of the breast cancer subgroup (RR = 0.88, 95% CI: 0.82 to 0.94), and in the subgroup with more than 20000 numbers of participants (RR = 0.87, 95% CI: 0.80 to 0.94). Despite the association not reaching statistical significance in some subgroups, there were no significant differences among these subgroup analyses (*P* > 0.05).

### 3.5. Metaregression Analyses and Publication Bias

**Figure 6:**
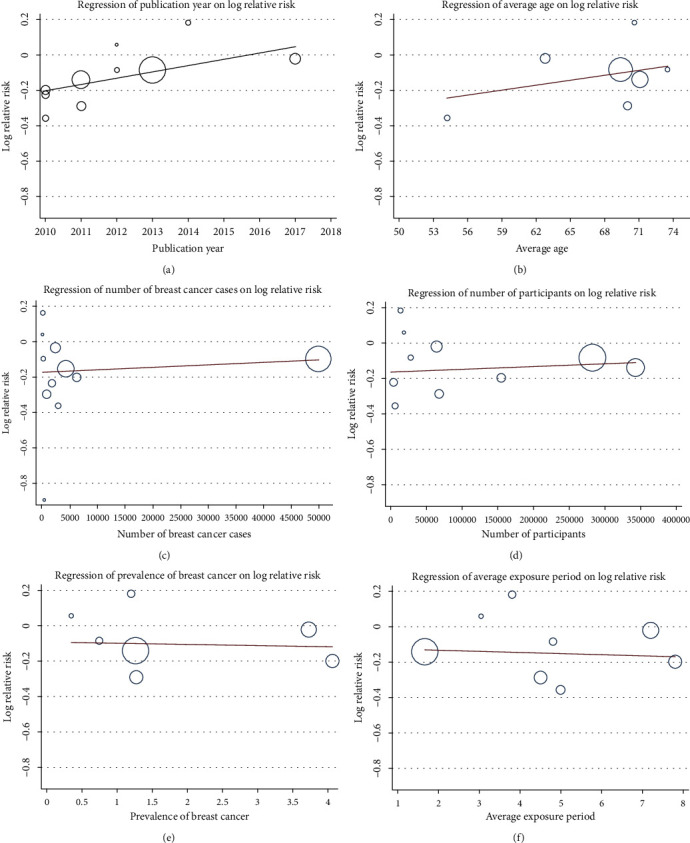
Random-effect metaregression analysis showing the relationship between the study effect size and (a) publication year, (b) average age, (c) number of breast cancer cases, (d) number of participants, (e) prevalence of breast cancer, and (f) average exposure period. The size of the circles is inversely proportional to the size of the result study variance, so that more precise studies have larger circles.

To investigate the latent reasons for the heterogeneity, we conducted a priori metaregression analysis. The scattered plots are graphically shown for the metaregression analysis (Figure 6). Metaregression analysis was statistically significant (*P* = 0.025) for the publication year in individual studies. While the average age (*P* = 0.433), number of breast cancer cases (*P* = 0.597), number of participants (*P* = 0.687), prevalence of breast cancer (*P* = 0.888), and average exposure period (*P* = 0.865) did not explain the demonstrated heterogeneity (Table S2). The main heterogeneity source may be from the publication year. There was no evidence of publication bias for the primary outcome by inspection of the funnel plot (Figure S2).

## 4. Discussion

The present meta-analysis summed up the results of four case-control studies and seven retrospective cohort studies, including a total of 69693 breast cancer cases. Usage of bisphosphonates significantly decreased the risk of breast cancer, with a RR of 0.87 (95% CI: 0.80–0.94), including contralateral breast cancer (RR = 0.41, 95% CI: 0.20 to 0.84). This protective efficacy of bisphosphonates was noted in women who taken bisphosphonates with long-term use (>1 year). The benefit varied between different types of bisphosphonates, specifically etidronate, which significantly lowered breast cancer risk by 13%.

Our results generally conformed with two previous meta-analyses [17, 18] that observed a 15% risk reduction in breast cancer after any use of bisphosphonates in four studies [6, 8, 13, 14] and a 16% reduction in breast cancer risk among bisphosphonate users in eight studies [6–8, 12–16]. Our analysis was based on a larger sample size, with a prior-registered protocol on PROSPERO, which may reinforce the quality and transparency of meta-analyses. Notably, obvious heterogeneity was observed in the present study; we conducted a sensitivity analysis by serial exclusion of individual studies. After eliminating one trial [12], it could only account for the partial heterogeneity. To further investigate other factors to study heterogeneity, we conducted a prior metaregression analysis. Our results indicated publication year may be the main source of heterogeneity with a descending trend on effect size. Remarkably, the included studies were performed with a wide range of study periods, during which the administration of breast cancer has altered to a great extent. The National Comprehensive Cancer Network (NCCN) Clinical Practice Guidelines with an update every few years in breast cancer (screening and diagnosis) [23] contributed to standardizing and improving administration of breast cancer, including early detection, early diagnosis, and early treatment. Over time, more and more new research failed to reveal a significant clinical efficacy from bisphosphonates, whereas a reduction in incidence of breast cancer was observed in older studies. Allegedly, in view of management as a part of a more standardized and comprehensive intervention, the effectiveness of bisphosphonates was blunted.

Structurally, bisphosphonates are categorized into older first-generation non-nitrogen-containing moieties and newer second-generation nitrogen-containing moieties, including etidronate, clodronate, and tiludronate as well as pamidronate, alendronate, ibandronate, risedronate, and zoledronate. Our meta-analysis found that only etidronate, not alendronate, showed a protective efficacy on the risk of breast cancer, in contrast with a published meta-analysis [18] in which nonnitrogen bisphosphonates (etidronate) and nitrogen bisphosphonates (alendronate) were associated with reduction of breast cancer risk (*d* rate, 13% and 9%, respectively) and in conformity with the Early Breast Cancer Trialists' Collaborative Group meta-analysis [24] in which subgroup analyses confirmed greater effectiveness with clodronate than with amino bisphosphonates on breast cancer mortality. Theoretically, nitrogen bisphosphonates containing nitrogen possess higher potency in anti-invasive, antiproliferation, and inhibiting angiogenesis than nonnitrogen bisphosphonates [25]. It is not clear why more potent nitrogen bisphosphonates appear to fail to produce a better beneficial outcome in breast cancer. On account of limited studies with small sample size, the present finding that etidronate has a protective effect on breast cancer risk may be insufficient to show a significant trend. To date, there are no researches focused on this topic. To comprehensively understand the certain bisphosphonates contributing to the antitumor effect, future larger prospective studies adequately powered are awaited to clarify issues.

In our meta-analysis, the antitumor effectiveness of the usage of bisphosphonates on breast cancer risk was observed only after more than 1 year of receiving bisphosphonates, which was also seen in two previous meta-analyses focusing on breast cancer [17, 18]. Interestingly, bisphosphonate users with other cancers, such as colorectal cancer and endometrial cancer [26, 27], also found a similar time-dependent beneficial efficacy. In a meta-analysis for colorectal cancer [26], participants who had taken bisphosphonates with a duration of 1 to 3 years (OR = 0.76, 95% CI: 0.68 to 0.85) and more than 3 years (OR = 0.78, 95% CI: 0.61 to 0.99) showed a significant inverse association, whereas those who used bisphosphonates for at least 1 year (OR = 0.91, 95% CI: 0.77 to 1.07) did not experience a beneficial efficacy. Another meta-analysis focusing on the risk of endometrial cancer [27] had a 43% risk reduction for endometrial cancer with long-term usage of bisphosphonates (>1 year) (RR = 0.69, 95% CI: 0.62 to 0.77), but not for short-term usage of bisphosphonates (<1 year) (RR = 0.89, 95% CI: 0.61 to 1.30). Furthermore, our findings indicated that exposure time association between bisphosphonates and the risk of breast cancer has an approximately linear trend. With regard to whether use of bisphosphonates with long term is actually beneficial for breast cancer risk, the side effect profile should be considered. Adverse effects of bisphosphonate users have been reported, including osteonecrosis of the jaw, atypical femoral fractures, atrial fibrillation, gastrointestinal disorders, and renal events [28, 29]. Therefore, the adverse effects of long-term use of bisphosphonates need to be analyzed in further prospective studies, to determine whether the benefits of therapy really outweigh the risks and to optimize bisphosphonate usage time.

In terms of the efficacy of bisphosphonates for different types of breast cancer, our risk-stratification analysis suggested that bisphosphonates have anti-breast cancer effects only in contralateral breast cancer (reduced rate, 59%), rather than in invasive breast cancer and DICS, inconsistent with a previous meta-analysis [18] in that bisphosphonate can lower incidence of invasive breast cancer, with a RR of 0.78 (95% CI: 0.68 to 0.91). Similar results were demonstrated in an observational cohort from Fournier et al. [9] wherein there was no association of bisphosphonate use with either in situ or invasive breast tumors. It is important to note that there are limited original studies that integrate subgroups, such as invasive breast cancer group (three studies), DICS group (two studies), and contralateral breast cancer group (one trial), so further investigation is required in this direction.

Meanwhile, this meta-analysis was also inevitably associated with several limitations, as shown below. First, marked heterogeneity existed across the included studies in terms of nonuniform study design, population characteristics, and definitions of exposure bisphosphonates. However, using sensitivity and metaregression analyses, we have found the sources of potential heterogeneity between studies. Second, though it was attempted to adjust for potential confounding factors in each eligible trial, a meta-analysis is not able to resolve residual or unknown confounders that could be inherent in the included studies. Third, due to lack of sufficient data, we cannot perform further subgroup analysis in terms of intake form (daily use vs. once a week use). Final, after cessation of treatment, we were not able to evaluate the period of time on whether the protective efficacy of bisphosphonate is lost or not. Further well-designed researches are warranted to resolve these issues.

## 5. Conclusion

Taken together, the results from this meta-analysis found that exposure to bisphosphonates was associated with decreased breast cancer incidence, including contralateral breast cancer. Compared with other types of bisphosphonates, only etidronate showed a significant inverse relationship. In additional, the long-term use (>1 year) of bisphosphonates was more significant in reducing the risk of breast cancer. Further in-depth longitudinal researches are urgently desirable to generate more precise estimates considering all potential confounders, different types of bisphosphonates, cumulative dose, duration of bisphosphonates use, and effects of bisphosphonates on different types of breast cancer.

## Figures and Tables

**Table 1:** Baseline characteristics of the studies included in the meta-analysis.

Study (year)	Nation	Study design	Period	Study database	Age (y), average (range or SD)	Menopausal status	No. of cases	No. of participants	Prevalence of breast cancer (%)
Cardwell (2011) [6]	UK	Cohort	1996-2006	GPRD	70 (11.4)	Pre- & post-	870	68098	1.28%
Chiang (2012) [7]	Taiwan	Cohort	1998-2009	NHIRD	73.5 (8.4)	Post-	209	27903	0.75%
Chlebowski (2010) [8]	USA	Cohort	1993-1998	WHI	NA (50-79)	Post-	6276	154768	4.06%
Fournier (2017) [9]	France	Cohort	2004-2011	E3N	62.8 (6.4)	Post-	2407	64438	3.73%
Hue (2014) [10]	USA	Cohort	1993-2006	FIT & HORIZON-PFT	70.6 (5.8)	Post-	165	13774	1.2%
Lee (2012) [11]	Taiwan	Cohort	1996-2009	NHI	NA (≥65)	Pre- & post-	65	18499	0.35%
Monsees (2011) [12]	USA	Case-control	1990-2007	SEER	NA (40-79)	Pre- & post-	351	1013	NA
Newcomb (2010) [13]	USA	Case-control	2003-2006	WMCR	54.2 (8.9)	Pre- & post-	2936	5911	NA
Rennert (2010) [14]	Israel	Case-control	2000-2006	CHS	63.6 (NA)	Post-	1832	4039	NA
Vestergaard (2011) [16]	Denmark	Cohort	1996-2006	DPB	71.1 (10.7)	Pre- & post-	4349	342651	1.27%
Vinogradova (2013) [15]	UK	Case-control	1997-2011	QResearch & CPRD	69.4 (9.8)	Pre- & post-	49933	282713	NA

Abbreviations: CHS: Clalit Health Service; CPRD: Clinical Practice Research Datalink; SEER: Surveillance, Epidemiology, and End Results; WMCR: Wisconsin Mandatory Cancer Registry; DPB: Danish population base; GPRD: General Practice Research Database; NHIRD: National Health Insurance Research Database; WHI: Women's Health Initiative; E3N: Etude Epidémiologique Auprès de Femmes de la Mutuelle Générale de l'Education Nationale; FIT: Fracture Intervention Trial; HORIZON-PFT: Health Outcomes and Reduced Incidence with Zoledronic Acid Once Yearly-Pivotal Fracture Trial; NHI: National Health Insurance; NA: not available; SD: standard deviation.

**Table 2:** Bisphosphonate use of the studies included in the meta-analysis.

Study (year)	Type of breast cancer	Type of BPs	Definition of exposure	Measure of BP use	Average exposure period (years)	Adjustment for covariates
Cardwell (2011) [6]	Breast cancer	BP	One or more prescriptions of oral BPs	Medical record	4.5	Smoking, alcohol, BMI, HRT, oral steroids, NSAIDs, calcium, and vitamin D
Chiang (2012) [7]	Breast cancer	ALN	Prescriptions of oral ALN	Medical record	4.8	Age, hypertension, diabetes, COPD, estrogen, dyslipidemia, CKD, CAD, colorectal polyp, benign breast disease, obesity, and statin use
Chlebowski (2010) [8]	Invasive breast cancer & DCIS	BP	One or more oral BPs	Self-report	7.8	Age, ethnicity, smoking, alcohol, physical activity, BMI, mammogram, estrogen, progesterone, calcium, vitamin D, hip fracture, and Gail 5 y risk of breast cancer
Fournier (2017) [9]	Invasive breast cancer & DCIS	BP	One or more oral BPs	Self-report	7.2	Age, BMI, time since menopause, HRT, calcium, and vitamin D
Hue (2014) [10]	Breast cancer	ALN & ZA	Oral ALN or ZA	Medical record	3.8	Age, BMI, smoking, ethnicity, age at first pregnancy, and family of breast cancer history
Lee (2012) [11]	Breast cancer	ALN	Prescriptions of oral ALN	Medical record	3.04	Age
Monsees (2011) [12]	Contralateral breast cancer	BP & ALN	One or more BPs	Medical record	≥1	Progesterone receptor, status of the first primary breast cancer, height, weight, BMI, diagnosed with osteoporosis or osteopenia, mammography, therapies received or first breast cancer, chemotherapy, radiation, parity, menopausal status, alcohol, smoking, HRT, and first-degree family history of breast cancer
Newcomb (2010) [13]	Invasive breast cancer	BP	One or more BPs	Self-report	5	Age, parity, age at first live birth, family history of breast cancer, BMI, menopausal status, age at menopause, type of hormone use, mammography, osteoporosis, smoking, height, HRT, and self-reports of medication use
Rennert (2010) [14]	Breast cancer	BP & ALN	One or more prescriptions of oral BPs	Medical record	5	Family of breast cancer history, age, sports activity, Jewish ethnic group, fruit vegetable consumption, BMI, statin, aspirin, calcium, vitamin D, HRT, red meat consumption, number of pregnancies, months of breast feeding, and age at first pregnancy
Vestergaard (2011) [16]	Breast cancer	ALN, CLN, ETN, & RIN	One or more dispensations of oral BPs	Medical record	NA	Use of systemic hormone therapy, irradiation, chemotherapy, and alcoholism
Vinogradova (2013) [15]	Breast cancer	BP, ALN, ETN, & RIN	One or more prescriptions of oral BPs	Medical record	1.67	BMI, smoking, alcohol, ethnicity, comorbidity, and use of other drugs

Abbreviations: BPs: bisphosphonates; DCIS: ductal carcinoma in situ; ALN: alendronate; ZA: zoledronic acid; CLN: clodronate; ETN: etidronate; RIN: risedronate; NA: not available; BMI: body mass index; HRT: hormone replacement therapy; NSAIDs: nonsteroidal anti-inflammatory drugs; COPD: chronic obstructive pulmonary disease; CKD: chronic kidney disease; CAD: coronary artery disease.

**Table 3:** Sensitivity and subgroup analyses for the association between any bisphosphonate use and risk of breast cancer.

Analysis	Categories		No. of studies	RR (95% CI)	*P* value	*P* values between subgroup
Sensitivity analysis	Adjusted confounders	≥2	10	0.86 (0.80-0.94)	0.0003^∗^	
Subgroup analysis	Study location	Western country	9	0.86 (0.79-0.94)	0.0004^∗^	0.49
		Eastern country	2	0.96 (0.71-1.27)	0.75	
	Study design	Cohort	7	0.89 (0.81-0.98)	0.02^∗^	0.26
		Case-control	4	0.78 (0.64-0.96)	0.02^∗^	
	Menopausal status	Pre- & post-	6	0.83 (0.75-0.93)	0.0008^∗^	0.27
		Post-	5	0.92 (0.81-1.04)	0.18	
	Measure of BP use	Medical record	8	0.87 (0.80-0.96)	0.004^∗^	0.75
		Self-report	3	0.85 (0.70-1.01)	0.07	
	Study quality	High quality	5	0.85 (0.63-1.15)	0.3	0.87
		Low quality	6	0.88 (0.82-0.93)	<0.0001^∗^	
	No. of cases	≤1000	5	0.87 (0.66-1.14)	0.31	0.93
		>1000	6	0.88 (0.82-0.94)	0.0001^∗^	
	No. of participants	≤20000	5	0.83 (0.63-1.08)	0.17	0.66
		>20000	6	0.87 (0.80-0.94)	0.0004^∗^	

Abbreviations: RR: relative risk; CI: confidence interval; BPs: bisphosphonates. Note: ^∗^*P* < 0.05.

## Abbreviations

RR: Relative risk
CI: Confidence interval
WHI: Women's Health Initiative
NHIRD: National Health Insurance Research Database
MOOSE: Meta-Analysis of Observational Studies in Epidemiology
OR: Odds ratio
HR: Hazard ratio
NOS: Newcastle-Ottawa Scale
DCIS: Ductal carcinoma in situ
MRA: Metaregression analysis
NCCN: National Comprehensive Cancer Network
CHS: Clalit Health Service
CPRD: Clinical Practice Research Datalink
SEER: Surveillance, Epidemiology, and End Results
WMCR: Wisconsin Mandatory Cancer Registry
DPB: Danish population base
GPRD: General Practice Research Database
E3N: Etude Epidémiologique Auprès de Femmes de la Mutuelle Générale de l'Education Nationale
FIT: Fracture Intervention Trial
HORIZON-PFT: Health Outcomes and Reduced Incidence with Zoledronic Acid Once Yearly-Pivotal Fracture Trial
NHI: National Health Insurance
NA: Not available
SD: Standard deviation
BPs: Bisphosphonates
ALN: Alendronate
ZA: Zoledronic acid
CLN: Clodronate
ETN: Etidronate
RIN: Risedronate
BMI: Body mass index
HRT: Hormone replacement therapy
NSAIDs: Nonsteroidal anti-inflammatory drugs
COPD: Chronic obstructive pulmonary disease
CKD: Chronic kidney disease
CAD: Coronary artery disease
REC: Representative of exposed cohort
SNC: Selection of nonexposed cohort
AE: Ascertainment of exposed
AOI: Absence of outcome of interest

## Supplementary Materials

Supplementary 1 Table S1: quality assessment with the Newcastle-Ottawa Scale for studies.

Supplementary 2 Table S2: metaregression analysis for the possible sources of heterogeneity.

Supplementary 3 Figure S1: sensitivity analysis by excluding study by Monsees et al. [12].

Supplementary 4 Figure S2: assessment of publication bias using a funnel plot.
