# Supplementary material for: Association Use of Bisphosphonates with Risk of Breast Cancer: A Meta-Analysis
Source: Biomed Res Int. 2020 Oct 6;2020:5606573. doi: 10.1155/2020/5606573 (PMC7568169; doi:10.1155/2020/5606573)
Supplement: Supplementary 1 — Table S1: quality assessment with the Newcastle-Ottawa Scale for studies. [file 5606573.f1.docx]

**TableS1. Quality Assessment With Newcastle Ottawa Scale for studies**

| Study | Selection | | | | Comparability | Outcome | | |  |
| --- | --- | --- | --- | --- | --- | --- | --- | --- | --- |
|  | **REC** | **SNC AE** | | **AOI** | **Design and**  **Analysis** | **Assessment** | **Enough**  **Follow-up** | **Adequate**  **Follow-up** | **Score** |
| Cardwell  (2011) | **☆** | **☆** | **☆** | **☆** | **☆** | **☆** | **☆** | **☆** | **8** |
| Chiang  (2012) |  | **☆** |  | **☆** | **☆** | **☆** | **☆** | **☆** | **6** |
| Chlebowski (2010) | **☆** | **☆** | **☆** | **☆** | **☆** | **☆** | **☆** | **☆** | **8** |
| Fournier  (2017) | **☆** | **☆** | **☆** | **☆** | **☆** | **☆** | **☆** | **☆** | **8** |
| Hue (2014) | **☆** | **☆** |  | **☆** | **☆** |  | **☆** | **☆** | **6** |
| Lee (2012) | **☆** | **☆** | **☆** | **☆** | **☆** |  |  | **☆** | **6** |
| Monsees  (2011) |  | **☆** | **☆** | **☆** | **☆** | **☆** |  | **☆** | **6** |
| Newcomb  (2010) |  | **☆** |  | **☆** | **☆** | **☆** | **☆** | **☆** | **6** |
| Rennert  (2010) | **☆** | **☆** |  | **☆** | **☆** | **☆** | **☆** | **☆** | **7** |
| Vestergaard  (2011) | **☆** | **☆** | **☆** | **☆** | **☆** | **☆** |  | **☆** | **7** |
| Vinogradova  (2013) | **☆** | **☆** | **☆** | **☆** | **☆** | **☆** |  | **☆** | **7** |

**REC=Representative of Exposed Cohort , SNC=Selection of Nonexposed Cohort，AE=Ascertainment of Exposed, AOI=Absence of Outcome of Interest, star (✩) was allocated to a particular item when it was adequately reported and addressed. The item “comparability” could be allocated with a maximum of two stars. Dashes indicate this item was not adequately reported or addressed.**
