# Supplementary material for: Association Use of Bisphosphonates with Risk of Breast Cancer: A Meta-Analysis
Source: Biomed Res Int. 2020 Oct 6;2020:5606573. doi: 10.1155/2020/5606573 (PMC7568169; doi:10.1155/2020/5606573)
Supplement: Supplementary 2 — Table S2: metaregression analysis for the possible sources of heterogeneity. [file 5606573.f2.docx]

**TableS2. Meta-regression analysis for the possible sources of heterogeneity**

| Variances | Coefficient standard | Standard error | *P* value | 95% confidence interval |
| --- | --- | --- | --- | --- |
| Publication year | 0.0353 | 0.0132 | 0.025 | 0.0056-0.0651 |
| Average age | 0.0092 | 0.0108 | 0.433 | -0.0186-0.0370 |
| Number of breast cancer cases | 0.0001 | 0.0003 | 0.597 | -0.0007-0.0009 |
| Number  of participants | 0.0001 | 0.0004 | 0.687 | -0.0009-0.0011 |
| Prevalence of breast cancer | -0.0067 | 0.0453 | 0.888 | -0.1231-0.1098 |
| Average exposure period | -0.0063 | 0.0355 | 0.865 | -0.0903-0.0778 |
