# Supplementary figures and images for: Association Use of Bisphosphonates with Risk of Breast Cancer: A Meta-Analysis
Source: Biomed Res Int. 2020 Oct 6;2020:5606573. doi: 10.1155/2020/5606573 (PMC7568169; doi:10.1155/2020/5606573)

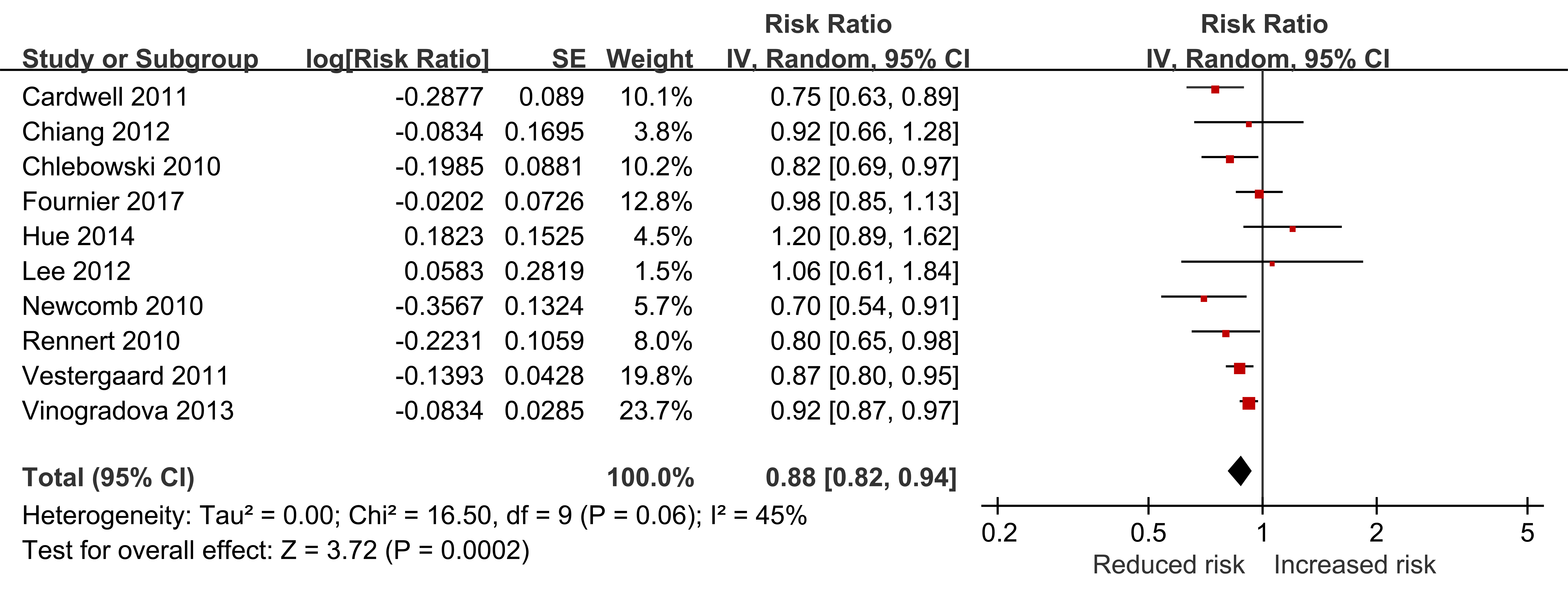

Supplement: Supplementary 3 — Figure S1: sensitivity analysis by excluding study by Monsees et al. [12]. [file 5606573.f3.tif]

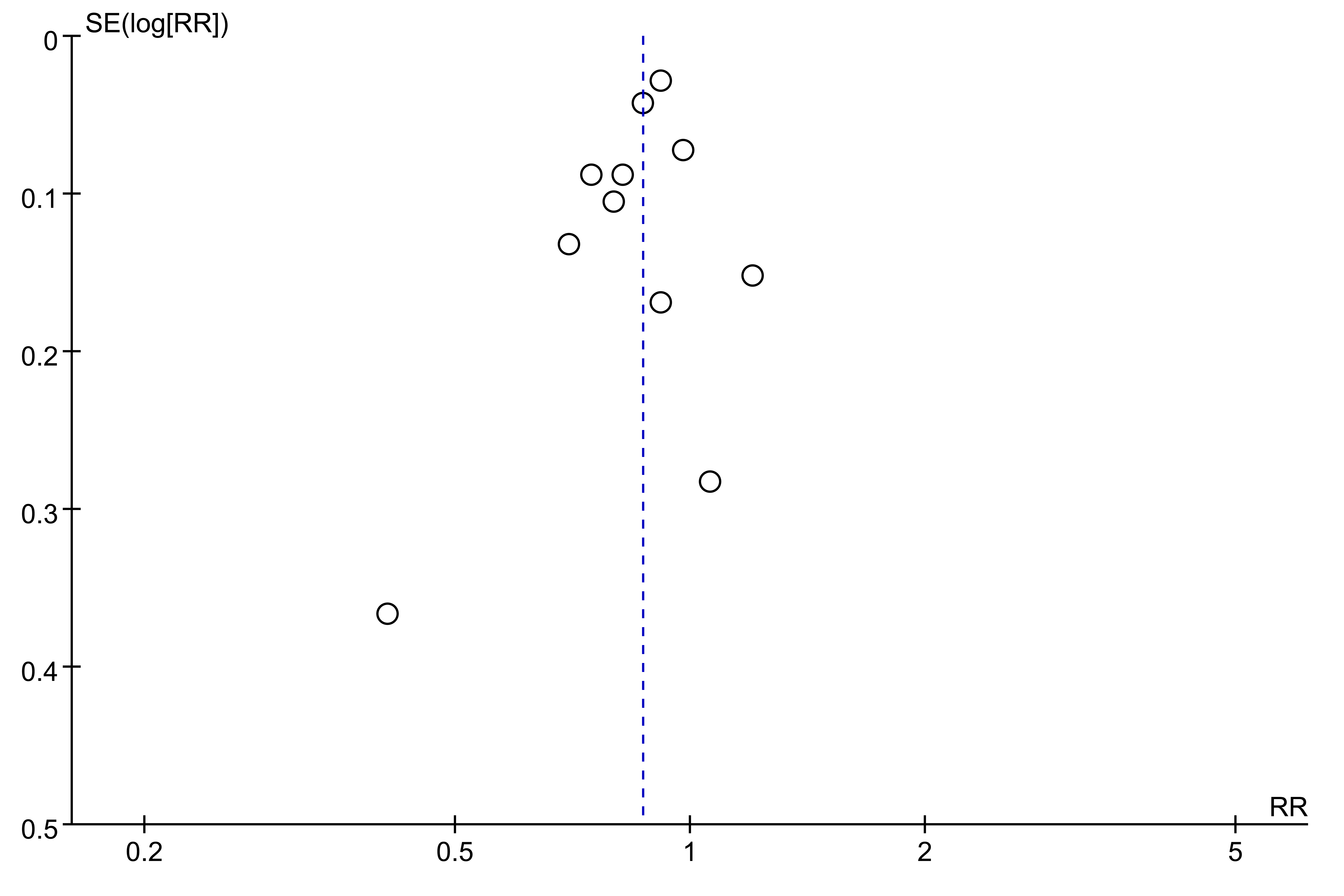

Supplement: Supplementary 4 — Figure S2: assessment of publication bias using a funnel plot. [file 5606573.f4.tif]
